# Examining Sentiment in Complex Texts. A Comparison of Different Computational Approaches

**DOI:** 10.3389/fdata.2022.886362

**Published:** 2022-05-04

**Authors:** Stefan Munnes, Corinna Harsch, Marcel Knobloch, Johannes S. Vogel, Lena Hipp, Erik Schilling

**Affiliations:** ^1^WZB Berlin Social Science Center, Berlin, Germany; ^2^Faculty of Economics and Social Sciences Chair of Inequality Research and Social Stratification Analysis, University of Potsdam, Potsdam, Germany; ^3^Institute for German Philology, Ludwig Maximilian University of Munich, Munich, Germany

**Keywords:** sentiment analysis, German literature, dictionary, word embeddings, automated text analysis, computer-assisted text analysis, scaling method

## Abstract

Can we rely on computational methods to accurately analyze complex texts? To answer this question, we compared different dictionary and scaling methods used in predicting the sentiment of German literature reviews to the “gold standard” of human-coded sentiments. Literature reviews constitute a challenging text corpus for computational analysis as they not only contain different text levels—for example, a summary of the work and the reviewer's appraisal—but are also characterized by subtle and ambiguous language elements. To take the nuanced sentiments of literature reviews into account, we worked with a metric rather than a dichotomous scale for sentiment analysis. The results of our analyses show that the predicted sentiments of prefabricated dictionaries, which are computationally efficient and require minimal adaption, have a low to medium correlation with the human-coded sentiments (r between 0.32 and 0.39). The accuracy of self-created dictionaries using word embeddings (both pre-trained and self-trained) was considerably lower (r between 0.10 and 0.28). Given the high coding intensity and contingency on seed selection as well as the degree of data pre-processing of word embeddings that we found with our data, we would not recommend them for complex texts without further adaptation. While fully automated approaches appear not to work in accurately predicting text sentiments with complex texts such as ours, we found relatively high correlations with a semiautomated approach (r of around 0.6)—which, however, requires intensive human coding efforts for the training dataset. In addition to illustrating the benefits and limits of computational approaches in analyzing complex text corpora and the potential of metric rather than binary scales of text sentiment, we also provide a practical guide for researchers to select an appropriate method and degree of pre-processing when working with complex texts.

## 1. Introduction

Quantitative text analysis has enabled researchers to process vast amounts of text in research designs of unprecedented size. Computational methods ranging from prefabricated, “off-the-shelf” dictionary approaches to fully automated machine learning approaches (Grimmer and Stewart, 2013) have been used to reliably analyze text corpora that are too large to read in a lifetime, including social media data (e.g., Twitter, Reddit), parliamentary debates, and online product reviews.

These new possibilities raise questions, however, about the validity and accuracy of computational methods used with different types of texts. While a given method may produce outstanding results for one text corpus, it may perform poorly on another. In this study, we therefore sought to answer the following question: Can computational methods also be used to predict the sentiment in linguistically complex texts—and if so, which methods should researchers choose to maximize accuracy and minimize costs? To assess whether and how accurately automated approaches can predict the sentiment of complex texts, we applied different methods to a corpus of reviews of contemporary German books, including both novels and non-fiction publications.

Book reviews constitute a challenging text type for computer-assisted text analysis. First, they tend to include different latent dimensions. In addition to a summary of the book's content, they contain the reviewer's judgment of the book. Sometimes they refer to other books or to current or past events. Second, the language used in reviewing books—novels in particular—itself tends to exhibit literary characteristics. Ambiguity, irony, and metaphors are difficult to capture, however, with automated approaches. Third, and closely related to the first two points, in contrast to texts that clearly express positive or negative assessments (e.g., product reviews), book reviews tend to lean in a positive direction. Low-quality books are either not reviewed at all or are criticized in cautious and ambiguous terms.

Our text corpus consists of a combination of a random sample and a purposive sample of book review summaries (N = 6,041) published on the German online literary magazine Perlentaucher. Based on this corpus, we compared the correlations between the sentiment that human coders identified in a given review (“the gold standard”) with the sentiment that different approaches predicted. Given the complexity and nuances of book reviews, we worked with a metric rather than a binary scale for sentiment analysis when applying different dictionary and scaling methods. In addition to prefabricated dictionaries (Remus et al., 2010; Rauh, 2018; Tymann et al., 2019), we also assessed the accuracy of self-created dictionaries based on word embeddings (GloVe: Pennington et al., 2014), and both supervised (wordscores: Laver et al., 2003) and unsupervised (wordfish: Slapin and Proksch, 2008) scaling methods. Given the importance of data pre-processing in computer-assisted text analysis, we also systematically varied the degree of text and dictionary manipulation when trying out the different methods to assess the influence on accuracy. With our analyses, we sought to provide guidance to other researchers in their decision-making processes for or against different methods.

The results of our comparison of the different approaches and different degrees of corpus pre-processing and dictionary modifications can be summarized as follows: First, prefabricated dictionaries, which are computationally efficient and require minimal, if any, adaption, such as the inclusion of negations, had a low to medium correlation with the human-coded sentiments (r between 0.32 and 0.39). Second, self-created dictionaries using word embeddings (both pre-trained and self-trained), which impose higher coding intensity on researchers, performed poorly with our corpus (r between 0.10 and 0.28). We would therefore not recommend them without further adaptations for complex text corpora similar to ours. Third, the fully automated approach we used in our analyses (wordfish) performed worst on our corpus, with correlations near 0. The semi-automated approach (wordscores), by contrast, which requires intensive human-coding of the training data, worked quite well. The correlations with the human-coded data ranged between 0.58 and 0.61 depending on the degree of pre-processing.

With these insights, our study makes the following contributions: First, we explore the potentials and limits of computational approaches for analyzing complex text corpora with regard to their validity and efficiency and provide researchers with a practical guide for selecting an appropriate method and the appropriate degree of pre-processing. Second, in contrast to most sentiment analyses, we work with a metric rather than a binary sentiment measure to take nuanced judgments into account, which may be beneficial for the analyses of many other complex text corpora as well. Third, we provide researchers, especially those working with non-English text corpora, with practical hints for creating context-specific dictionaries. Last but not least, by analyzing texts from outside the political arena, our analyses of a corpus of book reviews from contemporary German literature may inspire research projects outside established fields.

## 2. Background

### 2.1. Content Analysis in Times of Mass Communication

The analysis of text has always been of interest to social scientists. Words—both spoken and written—are an integral part of social realities and exert an enormous influence on individual behaviors and attitudes (e.g., Martin, 1991; Glasze, 2008; Klüver, 2009; Fisher et al., 2013; Walton and Boon, 2014; Ng and Leung, 2015). The major technique used to systematically extract data from different forms texts and classification of documents is content analysis. It is “a scientific tool” (Krippendorff, 2018, p. 18) to examine patterns in communication in a replicable and valid manner. Qualitative approaches to content analysis primarily rely on an interpretive understanding of meaning and semantic contexts; quantitative approaches, by contrast, use word frequencies, distributions, and statistics to classify texts. One of the key advantages of using content analysis to analyze social phenomena is its noninvasive nature, which sets it apart from approaches that simulate social experiences or collect survey answers. A major challenge for quantitative text analysis, on the other hand, is the variability of word meanings in different contexts.

The first content analyses were conducted at the beginning of the last century, when mass media had become a major communication tool, as a form of newspaper analysis. It became more relevant over the course of multiple economic crises and the two world wars as propaganda analysis (for the historical overview, see Krippendorff, 2018). After Berelson's (1952) characterization of quantitative content analysis as “a research technique for the systematic, objective, and quantitative description of the manifest content of communication” (p. 18), content analysis was applied to more and more research fields (for an overview, see Grimmer and Stewart, 2013; Benoit, 2020). In political science, quantitative content analysis has been used to study topics ranging from public discourse to individual policy positions and ideological networks. For instance, Glasze (2008) examined the discursive construction of Francophonie as a global community, international organization, and geocultural space. Stephens-Davidowitz (2014) analyzed how Google search terms can indicate racist animus and examined their impact on presidential elections in the United States. Similarly, Tumasjan et al. (2010) explored whether political sentiments on Twitter can predict election results (cf. critically Jungherr et al., 2012). Laver et al. (2003) and Diaz et al. (2016) assessed policy positions. Klüver (2009) and Sagarzazu and Klüver (2017) analyzed party manifestos, legislative speeches, interest groups in the EU, and political communication strategies of coalition parties. Fisher et al. (2013) analyzed discussions on climate change in the US Congress and mapped the resulting ideological relationships to measure coalitions and consensus among political actors.

In sociology, too, the benefits of using quantitative content analysis to study social phenomena has been recognized in recent years, and the method has been widely applied. Schwemmer and Wieczorek (2020), for instance, studied the methodological divide and paradigmatic preferences in sociology by analyzing publications in generalist sociology journals. Bohr and Dunlap (2018) applied topic modeling in their analyzes of sociological publications to identify the key topics in environmental sociology and changes in them over time. In their analysis of newspaper articles and Wikipedia entries, Nelson and King (2020) examined how distinct strategies emerge in different environmental organizations by linking their actions to their goals. In her analysis of US newspaper coverage on Muslim and non-Muslim women, Terman (2017) found more and different types of reporting on Muslim women than on non-Muslim women who had experienced human rights violations. Bail (2012) studied how civil society organizations shaped the news media discourse in the years after 9/11 through pro- and anti-Muslim messaging in their press releases.

Quantitative content analysis has also been used to investigate questions of social inequality in general and gender inequality in particular. In an analysis of Wikipedia profiles, Wagner et al. (2016) showed that women's profiles were more likely than men's to contain information on topics related to family, gender, and relationships and that the descriptions of men and women differed in the abstractness of positive and negative qualities. By analyzing men's and women's advertisements of their services in an online marketplace for contract labor, Ng and Leung (2015) showed that women were more likely to emphasize the relational aspects of their work, whereas men focused on the transactional aspects. Similarly, Hannák et al. (2017) analyzed worker evaluations from the online freelance marketplaces TaskRabbit and Fiverr and found considerable gender and racial biases in these evaluations. Brown (2021) analyzed descriptions of artworks to examine whether artworks produced by men and women differed in their observable characteristics and whether similarly described artwork by men and women varied in listing prices.

### 2.2. Sentiment Analysis in Digital Ages

According to Liu (2010), textual information can be “broadly categorized into two main types: facts and opinions” (p. 627). With sentiment analysis, which can be thought of a special form of content analysis and which has become one of the most important ways to quantitively analyze large amounts of textual data during the last 20 years, researchers seek to capture the nonfactual part of texts. Sentiment analysis, which is sometimes also referred to as “opinion mining” (Liu, 2012), captures the subjectivity, emotionality, or attitude of the author as expressed in the text; these are the aspects that are “not open to objective observation or verification” (Pang and Lee, 2008, p. 9). Sentiment analyses typically rely on dichotomous sentiment classifications (positive vs. negative) and sometimes also include a neutral category; there are, however, also studies that measured more nuanced emotional aspects, such as joy, anger, or sadness (Alm et al., 2005; Wiebe et al., 2005; Nielsen, 2011).

At the outset, sentiment analysis was mainly a subfield in computational linguistics and computer science. It's rise is mainly associated with the development of Web 2.0 in the early 2000s, which led to an incredible growth in the number of public available messages containing emotionally loaded opinions in form of product reviews, blog posts, forums contributions, or social media content. In addition, the big-tech-fueled commercialization of the internet has fostered a strong interest in the valorization of personal postings, as business models are built on the analysis of user behavior. Therefore, sentiment analysis has become widespread, especially in the financial and management sciences, but also in service, healthcare and the political and social sciences because of its importance to society as a whole; [(Liu, 2010; Puschmann and Powell, 2018); for an historic overview, also see Mäntylä et al. (2018)].

In contrast to classical quantitative content analysis methods, such as topic modeling or genre classification, in this method, the sentiments analyzed can be expressed in more subtle ways, including via the use of metaphors and irony. This makes sentiments much more difficult to detect (Pang et al., 2002). As a restricted natural language processing (NLP) problem, sentiment analysis does not need to understand the semantics of every sentence or the entire document but only some aspects of it. There are, however, two difficulties here: first, the task of determine the object to which the opinion is related and, second, the highly context-dependent nature of human language, which is especially true for evaluations (Liu, 2010). Ambiguity is also a problem in human coding, where coders do not always clearly come to the same conclusion about the subjective expression of opinion (van Atteveldt and Peng, 2018).

### 2.3. Various Computerized Methods

A key aspect of computerized sentiment analysis is that it is a tool to approximate human judgement. Obvious advantages of computerized methods include the reduced time and costs; researchers can thus deal with much larger corpora of texts (King, 2011). However, researchers have struggled with problems concerning the validity and accuracy of computerized methods compared to human judgment. For this reason, computerized coding is compared with the gold standard of manual coding of sentiment by human coders on different text with different languages, as we do in this article (Nelson et al., 2018; Puschmann and Powell, 2018; van Atteveldt et al., 2021).

Broadly speaking, the available computerized methods can be classified as first, prefabricated dictionaries, second, constructed dictionaries for specific contexts, and third, machine learning (Rudkowsky et al., 2018). Each of these methods comes with different advantages and disadvantages and presumably varies in their performance in accurately classifying texts or predicting text sentiment. See Table 1 for a general overview of the methods that will be discussed.

**Table 1:** Overview of various sentiment classification methods.

**Type**	**Method**	**Validity and reliability**	**Time and costs**
Gold standard	Human-coded	++	++
Dictionary	Prefabricated	−	−−
	Corpus-specific (e.g., word embeddings)	+	+
Maschine	Supervised (e.g., wordscores)	+	++
learning	Unsupervised (e.g., wordfish)	−	−−

One of the most common, intuitive, and feasible methods of measuring text sentiment entails the use of dictionaries. Dictionary methods use the appearance rate of certain words (or combinations of words) to measure specific characteristics of the text (Grimmer and Stewart, 2013, 274). Dictionaries usually contain a list of words with a certain score (i.e., negative or positive) attached to them (DiMaggio, 2015, 274). The frequency with which words in either one of these categories appears in a text document is then used to measure the polarity of this document. Prefabricated dictionaries impose low costs on researchers and are ideal for replication purposes. There are a number of dictionaries, in different languages, that are easy to download, and some are already included in common software packages.

The advantages of dictionary approaches are that they are easy to use, computationally efficient, reliable, and require minimal working time if prefabricated dictionaries are used. Some potential shortcomings of dictionary methods are that they lack specificity, sensitivity, and validity (Benoit, 2020, 14f.). That is, instead of associating all relevant words—and only those—with positive or negative sentiments, dictionary methods may identify content that is not relevant for classifying a text (a lack of specificity), may not identify all relevant content (a lack of sensitivity), or may identify content inaccurately (a lack of validity), as words can have multiple meanings (“polysemes”) and may be used differently in different contexts (e.g., in ironic discourse) (Grimmer and Stewart, 2013; Muddiman et al., 2019, 274). Dictionary accuracy may therefore vary depending on both the dictionary used and the characteristics of the text corpus. Recent advances in the development of multilingual (Proksch et al., 2019) and corpus-based dictionaries (Rice and Zorn, 2021) have sought to take these challenges into account.

Researchers can also modify prefabricated dictionaries according to their needs or engage in the tedious process of creating their own custom dictionaries (e.g., Muddiman et al., 2019) when the text under examination is very specific and uses unusual vocabulary and idioms (which may be the case with book reviews). Rice and Zorn (2021), for instance, have shown how to use certain machine learning methods to create a corpus-specific dictionary for specialized vocabularies in different contexts. The basic idea is to use what are known as word embeddings to find words that are similar to selected positive and negative words. Word embeddings are representations of words and their contextual meanings in a real-valued vector space. These specific methods of word embeddings are part of the broader field of natural language processing and refers to the distributional hypothesis proposed by Harris (1954). This hypothesis states that words appearing in the same context share the same meaning. Since this method creates word vectors using the global word-word co-occurrence statistics from a text corpus and neural networks, it is much more advanced and complex than dictionary approaches. However, it can be used for specific corpuses, and no human-coded training data is needed.

To overcome the challenges and shortcomings of dictionary approaches, researchers may also consider using either supervised or unsupervised machine learning methods, also known as classification and scaling methods (Grimmer and Stewart, 2013). Supervised machine learning methods require researchers to specify the relevant dimensions of interest in a set of pre-coded training texts, for example, the topic or the positive/negative text sentiment. Based on the dimensions specified in this training set, machine learning methods subsequently try to predict the characteristics of the unrated set of test texts (Benoit, 2020). Usually, such approaches entail attempting to classify the sentiment of a text into two or three categories. Classifiers like naive Bayes, maximum entropy or support-vector machines are used for this purpose. For our approach, which involves measuring sentiment in a more differentiated way on a metric scale, scaling methods are suitable.

A prominent supervised scaling method is wordscores (Laver et al., 2003). Wordscores assigns texts to a position on a continuous scale—the range of which is provided through the pre-coded training set. As is the case with dictionary methods, wordscores and other scaling methods have several advantages: replicability, reliability, speed, and low cost. Major disadvantages of supervised scaling methods are that the scaling of the texts in the training dataset requires considerable human coding for texts that are not yet classified. Moreover, the only words that are considered in the test dataset are those that were scaled in the training dataset, and only the relative importance of these words for determining the text sentiment is not contingent on the larger text content (similar to dictionary methods).

In terms of unsupervised scaling methods, wordfish (Slapin and Proksch, 2008) shares many of the advantages of wordscores but can be applied without reference texts and therefore requires less time and entails lower costs for researchers. However, the scale that unsupervised methods such as wordfish identifies may be unclear and corpus specific. As a result, it is difficult to replicate and compare the accuracy of text sentiment predictions across different corpora.

Regarding the current status of the general quality of the different methods, as of today, the “best performance is still attained with trained human or crowd coding” (van Atteveldt et al., 2021, p. 1). van Atteveldt et al. (2021) further conclude that neither dictionaries nor machine learning approaches “come close to acceptable levels of validity” (p. 1). While deep learning approaches outperform dictionary-based methods, they nonetheless fall short in comparison to human classification.

## 3. Data

### 3.1. Book Reviews as an Example of Complex Text

To investigate how accurately these different computational methods predict the sentiment in complex texts, we draw on a corpus of reviews of contemporary German books, including novels and non-fiction publications. Book reviews pose numerous challenges for automated analysis. First, book reviews commonly consist of various latent dimensions and linguistic elements. They usually comprise an overview of the plot that is formulated in relatively neutral terms, a contextualization of the work within the contemporary literary landscape, and an evaluation of the book by the reviewer. However, these dimensions are neither easily separated from each other, nor is the reviewer's assessment necessarily confined to the evaluation part. If, for example, reviewers see deficits in a book's structure, they will typically not summarize it in a neutral way. Reviewers may also judge a book differently depending on whether they approve of current literary trends. Second, book reviews are often characterized by linguistic ambiguities—ironic passages, metaphors, or sentences that praise a key idea but critique its realization. Third, book reviews often aim at surprising readers by creating certain expectations, only to subvert them and arrive at the opposite conclusion. In addition, reviewers may have various intentions, each with different implications: They may want to highlight a book's deficits or demonstrate their own broad knowledge. Hence, a neutral review that arrives at a matter-of-fact evaluation is more the exception than the rule.

In order to separate the different textual dimensions from each other and to reduce the text corpus to those passages in which reviewers provide their evaluation of the book, we decided not to work with the full-length reviews published in newspapers. Instead, we assembled our text corpus by collecting short versions of book reviews that focused on reviewer judgments from the German online literary magazine Perlentaucher, which has been in existence since 1999. Perlentaucher provides its readers with a daily overview of reviews published in the most important German newspapers and broadcast over the German public radio station Deutschlandfunk.

### 3.2. Data Collection and Sampling

The textual data of the summarized Perlentaucher reviews were collected along with additional information about the authors and books through web-scraping in May 2021.^1^ In total, 88,248 unique reviews of 54,744 books by 33,168 authors were collected. The mean number of reviews for the total of 51,126 books with at least one summary review on Perlentaucher is 2.44 (SD of 1.6). The median number of tokens (i.e., the building blocks of the text, which in our case are words) per review is 113, with 20 for the shortest and 932 for the longest review. For our analyses, we sought to reduce reviews of translations and non-fiction books in our sample.^2^

From this corpus, we first drew a random sample of more than 6,000 book reviews and supplemented these with a purposive sample of 612 additional reviews. The purposive sample consisted of books that were either very well or very poorly received, controversial, or widely debated in German feuilletons. This step of selection was supported by the literary experts we interviewed prior to data collection. The sample of randomly and purposively selected reviews was then used to establish the “true” sentiment of the short reviews—the “gold standard,” which we used to evaluate the accuracy of the different types of computational methods. In addition, we used a corpus containing all reviews with two different pre-processing strategies to train the word embeddings with the GloVe model.

### 3.3. Human-Coded Sentiment Analysis of Book Reviews

**Figure 1:**
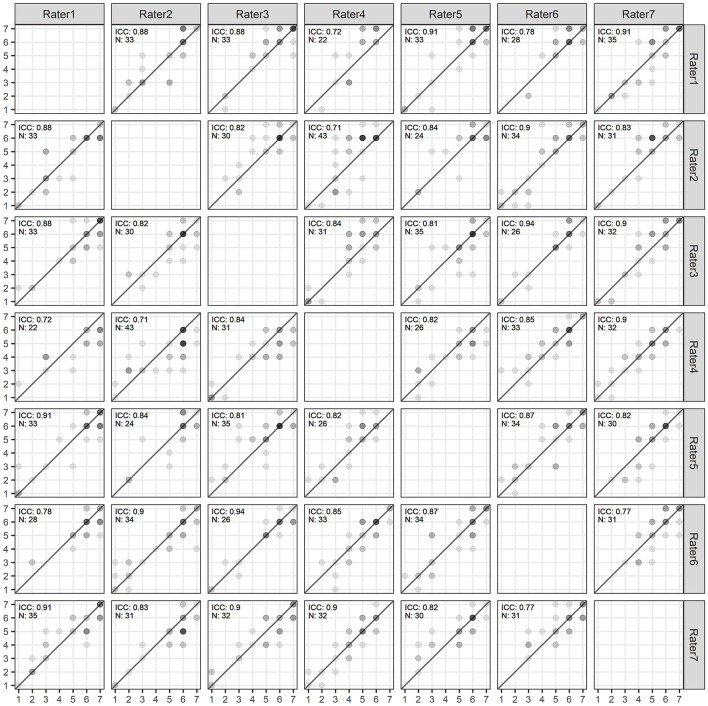
Scatter plot and ICCs of ratings between pairs of coders.

A total of seven paid, trained raters—most of them students with a background in literary studies—hand-coded the sentiment of the texts on a scale from 1 to 7 (very poor to very good)^3^ for 1,000 randomly drawn reviews^4^ per rater from the sample described above. After the completion of the coding process, we excluded reviews with missing scores and reviews that did not contain an evaluation. The final dataset of the human-coded reviews contained 6,041 valid sentiment scores. As expected, the reviews in our sample tended toward positive evaluations (median sentiment of 6, mean 5.09, and SD 1.66). Of these reviews, 656 were double-coded. We used these double-coded reviews to assess inter-coder reliability.^5^ The intraclass correlation coefficient (ICC) was 0.86 (95% CI: 0.84; 0.87). Figure 1 provides a scatter plot of inter-coder ratings. Based on the high consistency in the ratings (Liljequist et al., 2019), we assumed that all other reviews were also thoroughly and accurately coded. For reviews that were validly double-coded, we randomly chose one of two sentiment judgments for our analyses in order to have the same uncertainty measure in the evaluation.

### 3.4. Data Pre-processing

Data pre-processing is of vital importance for computational text analysis. Decisions about how to work with data should therefore always be made on the basis of pre-defined, methodological considerations (Denny and Spirling, 2018) as well as cost-benefit analyses associated with data cleaning and preparation. To enable researchers to make more informed decisions about the best degree of pre-processing for a given method, we examined how the accuracy of sentiment prediction of different methods varied between minimal and maximal levels of data pre-processing. The minimal pre-processing involved only the removal of punctuation, numbers, symbols, and separators from the reviews. The maximal pre-processing additionally involved the following alterations: We first stripped the reviews of the author names, the reviewer names, as well as the book titles and replaced all of them with empty tokens in order to maintain the original structure of the reviews. We then applied the same procedure to the tags and topics that had been assigned by Perlentaucher. These terms may affect how the different methods assess the sentiment of the reviews even if they are unrelated to reviewers' evaluations of the book (for example, in the case of the book *Ein schlechter Verlierer* or the author Freya Stark, the word “schlechter” (bad) and the last name Stark (also the word for strong) may influence the review sentiment). Third, we stemmed and converted all words to lowercase, changed all special German characters such as umlauts to Latin characters, and stripped the corpus of common stopwords. For this, we used the standard German stopwords list from the quanteda R package (Benoit et al., 2018) with two modifications: We deleted negating and strengthening words^6^ that may be important for sentiment detection and added review-specific words^7^ to it.

For the minimally pre-processed corpus, the median number of tokens per review was 115 (range 45 and 932) in our sample, that is, human-coded reviews; the median number of unique tokens was 92 per review (range 37–488). The reviews in the corpus with maximal pre-processing were much shorter for both tokens (median 56, range 19–536) and unique tokens (median 53, range 19–365). The extensive pre-processing hence indeed shortened the corpus substantially (reduction in median number of all and unique tokens by half) and reduced the number of words that occurred frequently and were presumably unnecessary to determine the text sentiment (shown by the small difference in the medians of all vs. unique tokens). Table 2 provides an illustration of how the original book review from Perlentaucher (column 1) changed with minimal data pre-processing (column 2) and maximal data pre-processing (columns 3).

**Table 2:** Illustration of minimal vs. maximal pre-processing on an examplary review.

**Original review**	**Tokens min. pre-processed**	**Tokens max. pre-processed**
“Rezensentin Christiane Pöhlmann freut sich zu früh über Literatur aus Lettland. Inga Abeles Roman dämpft ihr Leseglück doch recht schnell mit der Geschichte einer jungen Lettin zwischen dem drängenden Wunsch nach Selbstverwirklichung als Drehbuchautorin und Depression, die Pöhlmann zufolge einfach zu viel zwischen die Buchdeckel klemmen will, Perspektivwechsel, Monologe, Briefe, alternative Milieus, abstrakte Passagen über Lektüre, Exil und Russland. Die persönliche Tragödie der Protagonistin kommt darüber zu kurz, bedauert Pöhlmann.”	“Rezensentin” “Christiane” “Pöhlmann” “freut” “sich” “zu” “früh” “über” “Literatur” “aus” “Lettland” “Inga” “Abeles” “Roman” “dämpft” “ihr” “Leseglück” “doch” “recht” “schnell” “mit” “der” “Geschichte” “einer” “jungen” “Lettin” “zwischen” “dem” “drängenden” “Wunsch” “nach” “Selbstverwirklichung” “als” “Drehbuchautorin” “und” “Depression” “die” “Pöhlmann” “zufolge” “einfach” “zu” “viel” “zwischen” “die” “Buchdeckel” “klemmen” “will” “Perspektivwechsel” “Monologe” “Briefe” “alternative” “Milieus” “abstrakte” “Passagen” “über” “Lektüre” “Exil” “und” “Russland” “Die” “persönliche” “Tragödie” “der” “Protagonistin” “kommt” “darüber” “zu” “kurz” “bedauert” “Pöhlmann”	“” “” “” “freut” “” “” “frueh” “ueb” “literatur” “” “” “” “” “” “daempft” “” “leseglueck” “” “recht” “schnell” “” “” “” “” “jung” “lettin” “” “” “draengend” “wunsch” “” “selbstverwirklich” “” “drehbuchautorin” “” “depression” “” “” “zufolg” “einfach” “” “viel” “” “” “buchdeckel” “klemm” “” “perspektivwechsel” “monolog” “brief” “alternativ” “milieus” “abstrakt” “passag” “ueb” “lektu” “exil” “” “russland” “” “perso” “tragoedi” “” “protagonistin” “kommt” “darueb” “” “kurz” “bedauert” “”

## 4. Methods

In our comparison of how accurately different computational methods can predict the nuanced sentiments and evaluations of book reviews, we drew on the following approaches: First, we applied three prefabricated, German dictionaries to our corpus, namely SentiWS (Remus et al., 2010), Rauh's German Political Sentiment Dictionary Rauh (2018), and GerVADER (Tymann et al., 2019). Second, we applied a self-created, corpus-based dictionary to our corpus that we constructed using the GloVe algorithm by Pennington et al. (2014). Third, we applied a supervised (wordscores by Laver et al., 2003) and an unsupervised method (wordfish by Slapin and Proksch, 2008).

In contrast to the majority of common sentiment analyses, which only differentiate between a positive and a negative and sometimes also a neutral category, we used a metric sentiment scale for our analyses. We did this for two reasons. First, we wanted to do justice to the specificity of our text corpus: Book reviews are generally not either entirely good or entirely bad, but instead contain subtle distinctions in a wide range of judgments. Second, we wanted to stress-test the various methods and assess how well computational methods map onto the fine-grained differences in the evaluations. To ensure comparability, we therefore worked with z-standardized scales.

### 4.1. Prefabricated Dictionary Methods

The first dictionary we used in our analyses was SentimentWortschatz (SentiWS), which was developed by the Department of Natural Language Processing at the University of Leipzig (Remus et al., 2010). SentiWS contains a list of 15,559 negative and 15,491 positive words—adjectives, verbs, and nouns, as well as their inflections. These features make SentiWS well-suited for our two pre-processing approaches, as we did not manipulate the capitalization and inflections of words (which in German can change their meaning) in the minimally pre-processing approach.^8^

In our analyses, we applied the SentiWS dictionary to both the minimum and maximum pre-processed corpus, once without and once with modifications to the dictionary. The modifications reduced the number of positive and negative words to 2,343 and 2,575, respectively. To include negations in the modified SentiWS dictionary and match them with negations in our corpus, we followed Rauh's recommendation Rauh (2018) and replaced six pre-determined German negating terms^9^ with the English word “not” in our corpus. We connected the negating term with the following word as a bigram to form a single token that can be identified by the dictionary. To form the modified dictionary, we added a “not” negated version of each already existing token to the dictionary.

The second dictionary we used in our analyses was Rauh's German Political Sentiment Dictionary (Rauh, 2018), which is also available in the R package quanteda.sentiment (Benoit, 2021). The Rauh dictionary contains 74,160 entries, which are drawn from the SentiWS dictionary (Remus et al., 2010) and the GermanPolarityClues dictionary (Waltinger, 2010). In contrast to the two original underlying dictionaries, the Rauh dictionary also includes negated forms of each word. Accordingly, the entries are associated with four different keys: positive, negative, negated positive, and negated negative. To analyze the overall sentiment of a text, the negated positive words are meant to count as negative and the negated negative words as positive.

As with the other dictionary methods, we applied the Rauh dictionary to both the minimally and maximally pre-processed human-coded corpus. Similar to what we did in our analyses with the SentiWS dictionary, we replaced the negations in our text corpus with “not” and formed a bigram token. To compare the Rauh dictionary directly to the SentiWS dictionary, we also generated a minimally and maximally pre-processed version of the dictionary without the negated word forms. In the maximally pre-processed version, we performed the same steps as for the SentiWS dictionary: All words were stemmed, and German umlauts were transformed. This left us with a dictionary of 9,784 negative and 10,020 positive words in the dictionary containing negations. For the dictionary without negations, 6,161 negative and 4,028 positive entries were left.

The third dictionary we used in our analyses was GerVADER, a German adaption of the English language dictionary VADER (Hutto and Gilbert, 2014; Tymann et al., 2019). VADER consists of words taken from various other dictionaries such as the Linguistic Inquiry and Word Count dictionary (LIWC, Pennebaker et al., 2001) as well as special slang words and emoticons. The creators used crowd-coding to rate the polarity and intensity of each word. A strong feature of VADER are the heuristics implemented into the dictionary that allow a deeper understanding of text beyond bag-of-word analyses, in which the occurrence or frequency of words is used to classify texts, ignoring grammar or word order.^10^ VADER, moreover, includes intensifying adverbs, such as “extremely,” “very,” or “marginally,” and considers the mixed polarity of sentences starting with modifying conjunctions. VADER also examines trigrams preceding every word that carries sentiment and can therefore catch negations with a higher accuracy. VADER has been found to perform better in predicting text sentiment than other dictionary approaches and machine learning algorithms—and, in some instances, better than human coders (Hutto and Gilbert, 2014, 221).

The German VADER version, GerVADER, includes most of these features. The lexicon is based mainly on the SentiWS dictionary and was subsequently enlarged to include slang words. These words were then crowd-coded regarding polarity and intensity.^11^ GerVADER, however, does not perform as well as the original VADER English language dictionary—most likely due to lexical and grammatical differences between German and English that are not captured by a simple translation (Tymann et al., 2019, 11). In German, moreover, negating words often appear after the verb at the end of the sentence. As VADER only considers negating words before the sentiment-laden word, negated words tend to be detected less frequently in German language corpora. Furthermore, GerVADER struggles to correctly classify longer sentences.

As with the other dictionaries, we processed the GerVADER dictionary according to our minimal and maximal criteria. Most notable in this case was the stemming, which greatly reduced the words contained in the dictionary. The original GerVADER dictionary used for the minimal approach contained 16,477 negative and 18,020 positive words. After preparing for the maximal approach, the dictionary contained 3,331 negative and 4,072 positive terms.

### 4.2. Word Embeddings: GloVe

In addition to these prefabricated dictionaries (and their modifications), we created a corpus-specific dictionary by drawing on a machine learning algorithm. We followed the example of Rice and Zorn (2021) and used the GloVe algorithm (Pennington et al., 2014) to generate word vectors from our corpus to build a corpus-specific dictionary.^12^ We trained our own GloVe model, using the text2vev R Package (Selivanov et al., 2020), and created corpus-specific word embeddings. Here again, we varied the degree of pre-processing—this time for our total corpus of 88,248 reviews. For each pre-processed version, we also included a variant with additional bigrams in the word co-occurrence matrix to test whether negations and intensifications changed the results. For example, we wanted to see if word pairs like “not good” or “very good” would be part of the dictionary and would be attributed correctly.

There are various parameters in the modeling process that can be changed to identify the best model for a given dataset. For the purpose of our analyses, we followed the recommendations of Pennington et al. (2014) and Rodriguez and Spirling (2022). To have enough context for each token, we kept a minimum occurrence of five tokens. We also used a symmetric window size of 10, that is, five words before and five after the token. A larger window size (> 4) is recommended if the researcher is more interested in semantic than syntactic similarities. We also trained for the recommended 300 dimensions, the length of the resulting word vectors, with 10 iterations. This process resulted in four matrices of word vectors: The smallest is the maximum pre-processed variant with only onegrams (44,741 words and 105 MB of memory). The matrix with minimal pre-processing and onegrams contains 82,488 words and has a size of 194 MB. The matrix with maximal pre-processing and onegrams plus bigrams contains 95,674 words and has a size 226 MB. The matrix with minimal pre-processing contains 306,330 words and is 723 MB. On a computer with a CPU performance of 1.8 GHz and eight cores, the fitting of the models varied between 4 and 22 min.

As a next step, we used these four different matrices of word vectors to create our own dictionaries. This required positive and negative words as seeds to find similar words. To measure the similarity of the words represented as vectors, we used the cosine similarity. First, we used a list with 20 words, translated from (Rice and Zorn, 2021, henceforth RZ), which included generic and in principle interchangeable positive and negative terms, such as “brilliant” (brilliant), “wunderbar” (wonderful), and “schrecklich” (horrible). In a second step, we selected corpus-specific words from the hand-coded reviews that reflected the sentiment of the reviews, which we used as seeds (a total of 285 positive and 102 negative words, hence many more than in the first approach but including some very specific and rare words). These seeds were also pre-processed, so that they fitted the word vectors from the pre-processed corpus, which led to a seed corpus of 219 unique positive and 85 unique negative words for the maximally pre-processed corpus. In addition to typical words, these seeds also included words like “lustvoll” (lustful), “Poesie” (poetry), “Realismus” (realism), “Leichtigkeit” (easiness), or “kitschig” (cheesy), “billig” (cheap), “erwartbar” (expectable), and “Altherrenfantasie” (old men's fantasy).

We looped each list of seeds—both RZ's and the corpus-derived list—over the four word vector matrices. For each word in the dictionary, we collected the 400 words with the most similar vectors and kept words with a cosine similarity of at least 0.25. This relatively low similarity was a compromise between obtaining good similarity values and ensuring we had enough words to construct the final dictionary. In addition, only unique words that were not included in the other sentiment list were retained. Furthermore, only the same number of words per sentiment category was retained to avoid imbalance in the later matching process. Due to the exclusion of very rare words, the matrices of the word vectors no longer included all seeds. This resulted in a substantial variation of the dictionary length—from just 179 words per sentiment for maximum pre-processed and excluded bigrams with the RZ seeds to 1,017 for minimally pre-processed hand-coded seeds with bigrams included. See **Table 4** for an overview of the dictionaries along with the results.

Even if the first impression of this approach seemed to be promising, we also identified some conspicuous features of the resulting dictionaries that we consider worthwhile to briefly discuss. First, there were numerous words that, according to common understanding, do not express sentiments. The negative seed “Klischees” (clichés), for instance, yielded a list that included the non-evaluative word “Dimensionen” (dimensions) among others. Second, there were words with the exact opposite meaning from their seed. The word “Erstaunen” (astonishment), for example, was generated from the seed “Bedauern” (regret). Such mismatches were particularly likely to occur in the case of bigrams that involved negations. While bigrams such as “der_Stimulus” (the stimulus) or “gut_lesbar” (easy to read) yielded plausible lists of similar words, negations often fail to be assigned to the opposite negated sentiment.^13^

To further investigate the specific and relatively small corpus we used to train our GloVe models may mean that the results are not as good as a trained model on a larger corpus with much more contextual information for each word. We therefore also compared a pre-trained GloVe model with our model. The company deepset offers word vectors for free, trained with data from the German Wikipedia, which is a commonly used corpus for word embeddings due to its size. For pre-processing purposes, they only remove punctuation and lowercase, which is essentially the same as our minimally pre-processed corpus, and the minimal term frequency is also five. They also have a window size of 10,300 dimensions of vectors, and iterate 15 times. There are vectors for 1,309,281 words, much more than we achieve with our corpus. Because of the enormous number of words, we could let the minimum cosine similarity vary as a filter from 0.3 to 0.5 for both sources of seeds. Otherwise, we used the same procedure for selecting words. We obtained a dictionary size of 159 each for the RZ seeds and 322 for the human-coded ones for the most stringent selection of words with a cosine similarity of 0.5 to our seeds. With a cosine similarity of 0.3, the dictionaries contain 2,223 words each for the RZ seeds and 8,096 for the human-coded ones.

### 4.3. Scaling Methods: Wordscores and Wordfish

A third set of methods we used for our analyses were computational scaling methods, which have the advantage of being able to deal with very context-specific vocabulary. At the same time, they avoid much of the costly and labor-intensive preparation self-developed dictionaries require. Unlike methods using classification, the algorithms assign texts a position on a continuous scale (cf. Grimmer and Stewart, 2013, 292). Scaling methods are thus especially suitable for our approach, attempting to capture a more nuanced gradation of sentiment.

We used wordscores as an example of a supervised scaling method (Laver et al., 2003). We trained wordscores with the quanteda.textmodels R package (Benoit et al., 2021) with a training dataset that included around 50% of the human-coded reviews in our corpus (N = 3,015) and captured the entire range of all seven sentiments. The minimally pre-processed training data contained a total of 12,517 unique words and the maximally pre-processed data a total of 8,610 unique words.

The unsupervised machine learning method we applied to our corpus was wordfish, also included in the quanteda.textmodels R package. The algorithm was developed by Slapin and Proksch (2008) and goes a step further than wordscores as it does not require any human input. As an unsupervised machine learning approach, this scaling method assigns texts to positions on a scale entirely determined by the computer. This happens based on similarity in word use. The model builds on an assumed Poisson distribution of words across the corpus, from which it derives its name. With known word or document parameters, it could be calculated as a Poisson regression. Since both are unknown, two regressions are calculated alternately until they converge. Compared to wordscores, it thus has significant advantages: It does not require any human-coding or a human selection of reference texts. This maximizes the potential for reducing costs and labor. The downside is that, due to the scaling dimension being corpus-specific, it does not allow for any comparisons between analyses. Since the range is not determined by the researcher beforehand, the model is only able to capture the main dimension differentiating the texts. Wordfish has been able to work well with political left-right scales (Slapin and Proksch, 2008). Whether the easily replicable, reliable, and exceptionally cost-efficient scaling method does equally well with the subtle sentiment of complex literature reviews is the object of our test.

## 5. Findings

We now turn to the results of our analyses. In each of the sections below, we report the correlation between the human-coded sentiment of the reviews and the sentiment predicted by each method for the various levels of data pre-processing and degree of dictionary modification. In addition to reporting the substantive results in this section, we also develop recommendations for researchers interested in applying the different methods to complex text corpora.

### 5.1. Low to Medium Accuracy of Prefabricated Dictionary Methods

The accuracy of the different prefabricated dictionary approaches in predicting the sentiment of the book reviews is generally low, as can be seen from Table 3. First, the results of the SentiWS dictionary were not particularly good. Of the different pre-processing and dictionary variants, the lowest correlation was obtained with the maximally pre-processed approach that did not include negations (r = 0.29 with the human-coded sentiment). We were able to assign a sentiment for 6,033 out of the 6,041 human-coded reviews. On average, 8.55 words per review were matched with the dictionary content. To examine why SentiWS yielded a comparably low accuracy, we also counted the number of reviews whose predicted sentiment was completely off, that is, the deviation from the human-coded sentiment value was greater than two standard deviations. For the maximally pre-processed approach, this was the case for almost 552 reviews (10%). Under the condition of minimal processing, the correlation between the predicted and the human-coded sentiment value was slightly higher (r = 0.32) and results were further improved when negations were added (r = 0.38 with minimal pre-processing). After the inclusion of negations, however, only 6,012 reviews with an average of 6.34 matching words could be rated, and the number of ratings that were “completely off” also improved only slightly (427 reviews still had predicted sentiment values that were more than two standard deviations off; 7%). Based on these findings, we recommend adding additional negations to the SentiWS dictionary for the analysis of complex texts; other extensive pre-processing, however, may not be necessary.

**Table 3:** Characteristics and results for prefabricated dictionaries.

**Dictionary**					**Results**			
**Source**	**Negation**	**Pre-processing**	**# Pos**.	**# Neg**.	**N**	**Cor**.	**Matches** ^**a**^	**2 SD** ^**b**^
SentiWS		Minimal	15,591	15,559	6,033	0.32	8.55 (0.07)	552
		Maximal	2,343	2,575	6,031	0.29	8.88 (0.15)	540
	Negation	Minimal	31,150	31,150	6,012	0.38	6.34 (0.05)	427
	Negation	Maximal	4,918	4,918	6,033	0.36	9.23 (0.16)	421
Rauh		Minimal	17,330	19,750	6,038	0.39	9.38 (0.08)	429
		Maximal	4,028	6,161	6,041	0.37	16.00 (0.27)	439
	Negation	Minimal	37,080	37,080	6,035	0.39	8.23 (0.07)	422
	Negation	Maximal	10,020	9,784	6,041	0.36	15.10 (0.26)	483
GerVADER		Minimal	18,020	16,477	6,029	0.34	-	633
		Maximal	4,072	3,331	6,033	0.32	-	660

a *Average number (and share of average number of tokens) of tokens matched by the dictionary*.

b *Number of reviews that deviate more than 2 standard deviations from the human-coded results*.

Although the Rauh dictionary also performed rather poorly across all pre-processing variations in our corpus, it nonetheless yielded the second-best results of all the methods tested. With minimal pre-processing (both with and without negations), it achieved a correlation of 0.39 with the human-coded sentiment values. The original dictionary successfully determined the sentiment for 6,035 (6,038 without negations) reviews and matched a mean number of 8.23 (9.38) words per review on average. Moreover, the dictionary approaches with minimal pre-processing also performed better with regard to the number of predicted review sentiments that were more than two standard deviations away from the value that the human coders assigned (422 (7%) for the original dictionary with negations included and 429 (7%) for the dictionary with removed negations). We would therefore again recommend minimal pre-processing for the Rauh dictionary. Although including negations in the dictionary did not make sentiment determination considerably better, results did not deteriorate when the negated dictionary was combined with a minimal pre-processing approach. Since negations are already included in the Rauh dictionary, the extra step of excluding them was not worth the effort in our case.

Next, we turn to the results of the GerVADER dictionary. The results in Table 3 show that although GerVADER successfully scales most texts (*N* = 6,029 for the minimally and *N* = 6,033 for the maximally pre-processed corpus), correlations were only slightly better than the original SentiWS. For the minimal corpus, the correlation with human-coded results was 0.34, the correlation of the maximum approach was even lower (*r* = 0.31). It is not surprising that the maximum pre-processing had no positive effect on the dictionary, as GerVADER is more context-dependent than the other dictionaries included in our analyses. Interestingly however, the GerVADER dictionary underperformed compared to the negated SentiWS dictionary—presumably due to the higher number of predicted review sentiments that can be considered “completely off” (633–660; 10–11%). Although VADER is a promising tool for sentiment analysis, its German version may lack proper language implementation. It also needs to be noted that both the original VADER as well as GerVADER were originally intended for sentence-level classifications (in contrast to longer texts such as a book review) and were originally based on a 3-point classification (positive, negative, and neutral) and not on the more nuanced scale that we imposed and assumed for our corpus. Both issues may be additional explanations for its comparably poor performance.

### 5.2. Low Accuracy of the Work-Intensive Self-Created Dictionary Using Word Embeddings

The results of the self-created GloVe dictionary are shown in Table 4 and are neither good nor robust and vary greatly depending on seed selection and the degree of data pre-processing. Generally, the maximally pre-processed word vectors lead to better results than the minimally pre-processed vectors. The same applies to word vectors that do not contain bigrams. In terms of correlations, we observe slightly better and more consistent results with the human-coded seeds.

**Table 4:** Characteristics and results for self- and pre-trained GloVe dictionaries.

**Dictionary**						**Results**			
**Source**	**Seeds**	**Sim**.^**a**^	**Ngram**	**Preproc**.	**# P./N**.^**b**^	**N**	**Cor**.	**Matches** ^**c**^	**2 SD** ^**d**^
self-trained	hc	0.25		Minimal	425	5,748	0.21	3.77 (0.03)	683
		0.25		Maximal	257	5,779	0.28	3.76 (0.06)	575
		0.25	Bigram	Minimal	1,017	5,585	0.17	2.97 (0.02)	704
		0.25	Bigram	Maximal	269	5,823	0.24	4.04 (0.07)	695
self-trained	RZ	0.25		Minimal	317	6,038	0.17	11.11 (0.09)	747
		0.25		Maximal	252	6,004	0.26	6.16 (0.10)	589
		0.25	Bigram	Minimal	452	6,041	-0.01	15.19 (0.13)	964
		0.25	Bigram	Maximal	179	5,919	0.15	4.78 (0.08)	720
pre-trained	hc	0.3		Case ins.	8,096	6,041	0.15	36.18 (0.30)	772
		0.4		Case ins.	1,916	6,041	0.23	16.39 (0.14)	681
		0.5		Case ins.	322	5,963	0.26	5.01 (0.04)	573
pre-trained	RZ	0.3		Case ins.	2,223	6,041	0.10	31.54 (0.26)	886
		0.4		Case ins.	811	6,041	0.10	20.14 (0.17)	828
		0.5		Case ins.	159	5,958	0.10	5.89 (0.05)	803

a *Minimum cosine similarity of word vectors to each seed*.

b *Number of positive and negative words each*.

c *Average number (and share of average number of tokens) of tokens matched by the dictionary*.

d *Number of reviews that deviate more than 2 standard deviations from the human-coded results*.

The best results were obtained with the maximally pre-processed word vectors that did not contain bigrams. For the human-coded seeds, we observed a correlation of 0.28, and a correlation of 0.26 for the RZ seeds. The worst results were from the minimally pre-processed corpus with bigrams included. While a correlation of 0.17 was still achieved with the human-coded seeds, the RZ seeds yielded a value of –0.01. We also observed only 3–4 matches with the human-coded seed dictionaries, in comparison to 15 at the top for the smaller RZ dictionaries. It seems that the smaller but more specialized dictionary of human-coded seeds matches fewer words in the texts, but that these lead to a more accurate sentiment score, especially when the dataset was maximally pre-processed. The major downside to the more specialized, human-coded seed dictionaries was that no sentiment could be assigned for around 200 to 500 reviews.

For the pre-trained word vectors, we found the same pattern. Here, again, the dictionary with the corpus-specific seeds performed significantly better. While the dictionary derived from the RZ seeds had a constant correlation of only 0.1, when cosine similarity was increased from 0.3 to 0.5., the correlation for the dictionary derived from the corpus-specific seeds increased from 0.15 to 0.26. On average, only 5 words were matched for the best score, and about 80 reviews could not be scored at all for both maximally pre-processed dictionaries. The number of reviews that were incorrectly rated (> 2SD) was not as high as with the self-trained word vectors.

All in all, our self-created dictionaries based on word embeddings underperformed compared to the easier-to-implement, prefabricated dictionaries that we used on our corpus. If word embeddings are used to create dictionaries, we recommend the following: Better results can be achieved with a maximally pre-processed corpus; the additional use of bigrams does not improve the dictionary's accuracy. Self-trained vectors perform better than pre-trained vectors. Corpus-specific seeds lead to more accurate results than generic seeds. Furthermore, at least for the hand-coded seeds, a higher similarity of the words improves the results. In short, the more specific the words in the dictionary, the better the results.

### 5.3. High Accuracy of Semi-supervised but Low Accuracy of Unsupervised Scaling Methods

**Figure 2:**
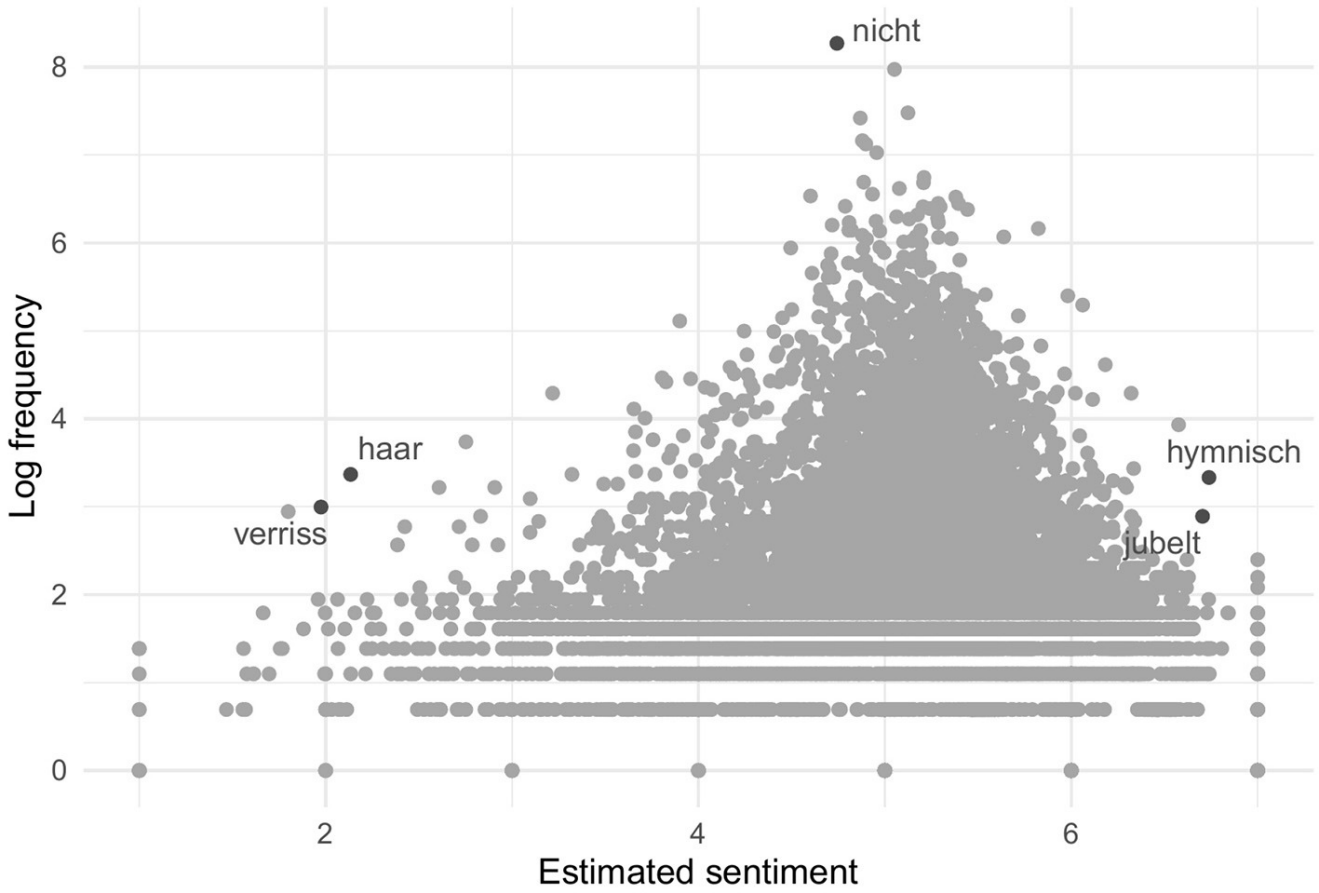
Sentiment of words estimated by supervised wordscores.

The wordscores algorithm calculated sentiment positions for 98.4% of the minimally and 99.4% of the maximally pre-processed words. Since the training texts were coded relatively positively with only a few clearly negative reviews, wordscores also yielded many more positive than negative words. With a threshold of 4 on the original 7-point scale, 11,124 minimally and 7,760 maximally pre-processed words can be considered positive and 1,193 minimally and 797 maximally pre-processed words negative. However, as Figure 2 illustrates, the actual attribution of sentiment is not binary but continuous: A word can also be only slightly more positive or negative than another. Words that occur frequently tend to be assigned a relatively neutral sentiment. This is not surprising, as a term that appears in both positive and negative reviews—for instance, pronouns or merely descriptive words—usually do not carry much clear sentiment. This is illustrated by the peak in Figure 2. Five frequent negative, neutral, and positive terms are highlighted as an example: “verriss” scorcher), “haar” (hair), “nicht” (not), “hymnisch” (anthemic), and “jubelt” (jubilates).

In the next step, the algorithm predicted the positions of the remaining 3,026 texts, based on the calculated ratings for the given words.^14^ Since the wordscores “dictionary” is rather comprehensive, it matches, in clear contrast to the previous actual dictionaries, 99.9% (minimal corpus: 100%) of the 119.5 (minimal corpus: 58.9) words per review in the estimation set on average. This may explain the moderate to strong correlation of the estimated sentiment of the texts with our human-coded results of 0.58 for the minimally and 0.61 for the maximally pre-processed corpus. This is the best result we achieved and is 0.2 points higher than with the best dictionary approach. In addition, only 76 (3%)–84 (3%) (minimally to maximally) texts were rated more than two standard deviations off (see Table 5). This confirms our initial assumption that our corpus uses very specific language that is not adequately captured by prefabricated dictionaries. The method is also more accurate than the word embeddings approach, since it evaluates more words accurately. However, the cost for this good result is the amount of human coding required for the training texts (50% of the corpus).

**Table 5:** Characteristics and results for supervised and unsupervised Methods.

**Source**	**Pre-processing**	**# Pos**.	**# Neg**.	**N**	**Cor**.	**Matches** ^**a**^	**2 SD** ^**b**^
Wordscores	Minimal	1,193	11,124	3,026	0.58	119.34	84
	Maximal	797	7760	3,026	0.61	58.91	76
Wordfish	Minimal	-	-	6,041	–0.05	119.50	1,095
	Maximal	-	-	6,041	–0.01	58.93	943

a *In contrast to dictionaries, almost all tokens (reported average) are used for scaling*.

b *Number of reviews that deviate more than 2 standard deviations from the human-coded results*.

**Figure 3:**
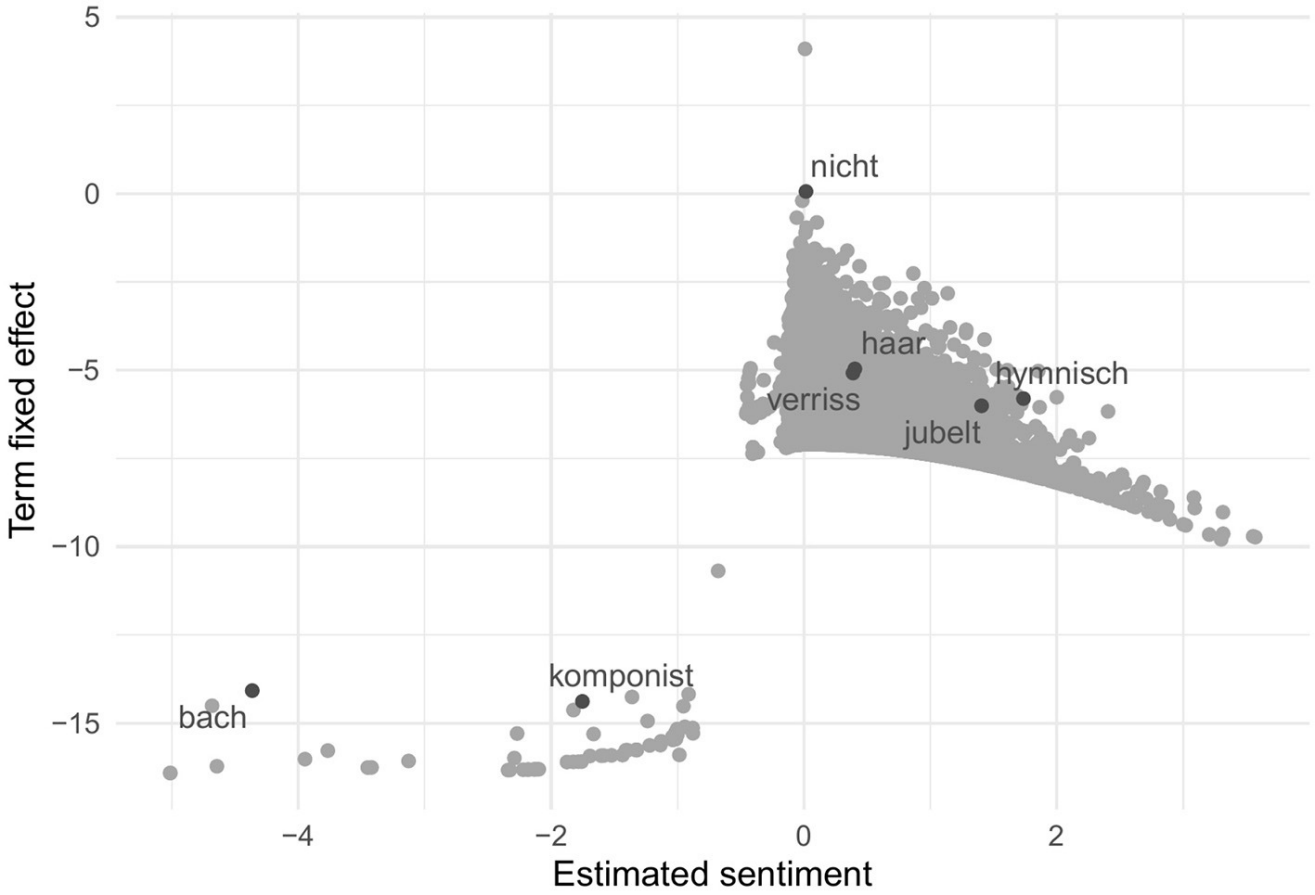
Sentiment of words estimated by unsupervised wordfish.

Without relying on any human input, the wordfish algorithm calculated sentiment positions for all 12,517 minimally and 8,610 maximally pre-processed words in the corpus. Since the resulting scale is metric and exceeds the original seven points, however, a dichotomization into positive and negative appears difficult. While the median could serve as a threshold, this would obscure the expected unequal distribution of more positive than negative terms. We therefore refer to Figure 3 to illustrate that the model has indeed converged and yields the expected Poisson distribution of words. The same five highlighted terms, however, already indicate that the estimation of sentiment was at most partially successful. While the words keep appearing in slightly different places, the opposing sentiment is no longer captured by the entirety of the scale.

Our doubts as to whether the wordfish estimation yields the sentiment of the reviews (rather than, for instance, the genre, the topic, or a mixture of these) grows when we compare the estimated sentiment positions of the texts with our gold standard, the human-coded results. While the unsupervised wordfish algorithm requires no human input for learning, estimates positions for all 6,041 texts, and matches 100% of the words in the estimation set, it yields a very weak correlation of –0.05 for the minimally and –0.01 for the maximally pre-processed corpus. In addition, 1,095 (18%) [minimal corpus: 943 (15%)] of texts were predicted more than two standard deviations off (see Table 5).^15^

Wordfish, despite its many advantages, is therefore not able to provide a useful sentiment estimation for our complex literature reviews. Since the algorithm only captures the least latent dimension, it appears that our text corpus is still too heterogeneous. For instance, some word positions point to a possibly involved dimension fiction–non-fiction, with a particular focus on music.^16^ With further controls, such as in the enhanced Wordshoal algorithm of Lauderdale and Herzog (2016), which allows for control of intervening variables, the model might therefore yield better results. Yet as the necessary additional information (e.g., literary genre) is not available reliably for our source of literature reviews, for our corpus and with the information at hand, we recommend supervised scaling or dictionary methods instead.

## 6. Conclusion

In this paper, we applied different computational text analysis approaches to a corpus of short summaries of German book reviews to examine whether different computational methods accurately predict the sentiment in complex texts—and if so, under what conditions. Examining these questions is important for several reasons. First, social scientists are working increasingly with text-as-data to analyze topics of great political and societal interest, such as changes in political and social discourse and communication strategies or the representation of minorities in newspapers and Wikipedia entries. With increasing text complexity and potentially also increasingly complex questions, it is crucially important that researchers are aware of the potentials and limits of the different approaches and choose computational methods that work best on their corpus. Second, assessing text sentiments in complex texts and capturing gradual differences—for example, in the description of certain groups—tends to require more than a binary assessment of whether a text is positively or negatively loaded. Instead, researchers may be interested in assessing degrees of positivity and negativity. Third, although the introductory literature on approaches to quantitative text analysis is constantly growing, researchers lack sufficient guidance on what degree of data pre-processing and modifications to existing tools is beneficial when using different approaches.

With our analyses, we sought to contribute to each of these important points. In addition to comparing how well different computational methods—including three prefabricated German language dictionaries (SentiWS, Rauh, GerVADER), a self-created dictionary using pre- and self-trained word-embeddings (GloVe), and one supervised and one unsupervised scaling method (wordscores and wordfish)—predict the sentiment of complex texts, we used a metric instead of a binary scale to assess text sentiment, and systematically varied the degree of data pre-processing for each approach.

According to our analyses, predefined German-language dictionaries showed average performance on our corpus. Relying on predefined dictionaries is easy and inexpensive; however, the simple counting of predefined, labeled words does not account for the specific contexts in which words are used or correctly identify special linguistic features, such as metaphors, irony, and allusions. Additionally, dictionary approaches cannot solve another general problem of content analysis—the detection of a sentiment's object. With dictionary approaches, it is impossible for the researcher to differentiate between the content, the evaluation, and further contextual information that is included in unstructured texts. Based on our findings, we recommend that researchers include negation terms when analyzing complex texts via these cost-efficient dictionary approaches.

Self-created dictionaries using word embeddings—both pretrained and self-trained—are a promising approach for analyzing texts for which predefined dictionaries were not designed. However, dictionary approaches using word embeddings impose high coding demands on researchers and actually performed poorly with our corpus. In theory, this approach intends to better capture the linguistic subtleties through the corpus-specific compilation of a list of words. When creating dictionaries based on word embeddings, researchers must deal with the trade-off between a small and highly specific dictionary and a large and unspecific dictionary by varying the cosine similarity to the chosen seeds. Although we sought to find a good compromise between a high similarity with the seeds and a sufficient number of words, with our corpus, the self-created dictionary was considerably less accurate in predicting the text sentiment than the prefabricated dictionaries. Furthermore, the results we obtained with word embeddings were not robust and varied considerably by seed selection and data pre-processing. Based on our experience, we suggest that researchers who apply the method manually should review the generated word lists and consider adding a small list of corpus-specific words to an existing dictionary.

There was considerable variation in the performance of the different machine learning approaches we applied. First, the accuracy in sentiment prediction based on wordfish, the unsupervised machine learning method, was even lower than the accuracy we obtained based on the prefabricated dictionaries. This low inaccuracy may be related to the many different latent dimensions that complex texts tend to have. In our text corpus, for instance, the content, genre, and evaluation of the book are all intermingled. The algorithm, however, only captures the least latent dimension. When using unsupervised scaling algorithms, researchers should try to reduce the number of text dimensions (which is a challenging task in unstructured texts, as was the case with ours). Second, the accuracy in sentiment prediction based on wordscores, the supervised machine learning method, was quite promising. The correlations between the predicted sentiment and the human-coded sentiments ranged between 0.58 (involving minimal data pre-processing) and 0.61 (with maximal data pre-processing). Given a sufficient number of classified texts, supervised learning methods fairly accurately identify patterns and predict the sentiment in even complex and specialized texts. The downside of the approach, however, is the high cost that the method entails in terms of the human coding necessary to train the model.

In conclusion, our results emphasize the importance of carefully choosing and evaluating different methods to ensure an optimal fit of the method to the data. Not only the methods used in the analyses but also the pre-processing influences the results, although not to a high and unambiguous degree. As a consequence, the research process should not be static, and the methods used should be constantly evaluated, adjusted, re-evaluated, and validated throughout the course of the project. In particular, by using word embeddings to create a corpus-specific dictionary, our results show both the potential and limits (as well as need for further advancements) of corpus-specific approaches. Overall, the analyses performed for this article provide researchers with some guidelines and ideas for how this can be done. In conclusion, we recommend scholars rely on supervised machine learning methods when resources are available. When resources are unavailable, scholars can implement certain protocols to help other methods perform better.

## Data Availability Statement

The datasets presented in this study can be found in online repositories. The names of the repository/repositories and accession number(s) can be found below: https://github.com/StefanMunnes/frontiers_literature/tree/master/data.

## Author Contributions

SM collected, organized, and cleaned the reviews database. MK and SM organized and supervised the sampling and hand coding process and wrote the section on data collection, manual coding, and pre-processing. CH, MK, and ES created a list of books for the purposive sample. CH and MK human-coded the seeds for the word embeddings. SM and JV pre-processed the data. Overall and final organization and cleaning of the code was done and coding and writing on embeddings and GloVe by SM. LH wrote the introduction and the conclusion and streamlined the article with support from SM. SM with help of the other authors contributed to the background section. Coding and writing related to the dictionaries was performed by SM, CH, and MK. Coding and writing on wordscores and wordfish by JV. Project management was the responsibility of LH, SM, and ES. LH and ES acquired the necessary funding. All authors proofread and approved the manuscript and participated in the conception and design of the study.

## Funding

This research was partly funded by Junge Akademie. The publication of this article was funded by the Open Access Fund of the Leibniz Association.

## Conflict of Interest

The authors declare that the research was conducted in the absence of any commercial or financial relationships that could be construed as a potential conflict of interest.

## Publisher's Note

All claims expressed in this article are solely those of the authors and do not necessarily represent those of their affiliated organizations, or those of the publisher, the editors and the reviewers. Any product that may be evaluated in this article, or claim that may be made by its manufacturer, is not guaranteed or endorsed by the publisher.

## Footnotes

^1^All R-scripts and important data for replication can be found at the GitHub repository.

^2^To exclude translations, we relied on the standard phrase in Perlentaucher book descriptions stating the language in which the book was originally published (“Aus dem LANGUAGE von …”) as well as books that Perlentaucher labeled as non-fiction (“Sachbuch”) in the tag or topic classification of the book. Moreover, we scraped additional Dewey Decimal Classification data from the German National Library in order to identify reviews of fiction books.

^3^Coders could also indicate if they were not able to find any evaluation of the book in the review at all; these reviews were coded as missing values and excluded.

^4^Book titles were randomly drawn so that all associated reviews would be rated by a single coder. There were an average of 2.44 reviews per book.

^5^Raters did not know how many and which reviews were also coded by another rater.

^6^For example, “aber” (but), “kein” (no), “sehr” (very), “viel” (much).

^7^For example, “Autor” (author), “Rezensentin” (reviewer), “Buch” (book).

^8^The original SentiWS dictionary also contains weights for the strength of sentiment for each word, but we only used the binary sentiment version provided in the quanteda.sentiment R package (Benoit, 2021) for better comparability with the other approaches.

^9^We added the word “ohne” (without) to Rauh's suggested list of “nicht” (not), “nichts” (nothing), “kein,” “keine,” and “keinen” (all inflections of the word no).

^10^For instance, VADER assigns higher scores to sentences ending with multiple exclamation marks or words that are written in all uppercase letters. This makes VADER especially useful for social media analyses, for which it was developed and for which it showed better results than other dictionaries. However, as our corpus was made up of reviews originally published in newspapers, the language is much more formal.

^11^It is important to note that, contrary to the original VADER, the raters did not receive financial compensation, which could have impacted their motivation and the data quality (Tymann et al., 2019, 6).

^12^We would like to point out that as of today, the word2vec algorithm (W2V), which was introduced by Google developers (Mikolov et al., 2013), is an additional, widely used and well documented algorithm that could be used for building a corpus-specific dictionary. W2V includes two different learning models: Continuous Bag of Words and Continuous Skip-Gram. While the first tries to predict every specific word based on a window of surrounding context words, the second tries exactly the opposite: It estimates the surrounding words from the specific word.

^13^Unfortunately, there is no simple way to pre-determine the quality of the choice of words in advance. We have deliberately chosen not to edit the dictionaries by hand, even though some ambiguities are clearly apparent. On the one hand, we assume that the meaning, which is partly not obvious to us, results methodically from the corpus. On the other hand, we would expect the wrong meanings to average out. Nevertheless, we assume that significant improvements could be made at this point in the procedure with some effort if the dictionaries were manually edited.

^14^The total runtime of the wordscores model was very moderate with 40–60 s per corpus.

^15^The total runtime of the wordfish model was 5–50 min per corpus.

^16^Features scaled as very negative, for example, were “bach” (river, but more likely the composer), “wohltemperiert” (well-tempered), “klavi[er]” (piano), “musikwissenschaft” (musicology), and “komponist” (composer).
